# Klotho Levels and Their Relationship with Inflammation and Survival among Alcoholic Patients

**DOI:** 10.3390/biom12081151

**Published:** 2022-08-20

**Authors:** Candelaria Martín-González, Elisa Espelosín-Ortega, Pedro Abreu-González, Camino Fernández-Rodríguez, Víctor Eugenio Vera-Delgado, Lourdes González-Navarrete, Alen García-Rodríguez, Antonio Martínez Riera, Emilio González-Reimers

**Affiliations:** 1 Departamento de Medicina Interna, Universidad de La Laguna, Servicio de Medicina Interna, Hospital Universitario de Canarias, Tenerife, Canary Islands, 38320 La Laguna, Spain; 2 Servicio de Laboratorio, Hospital Universitario de Canarias, Tenerife, Canary Islands, 38320 La Laguna, Spain; 3 Departamento de Ciencias Médicas Básicas, Unidad de Fisiología, Universidad de la Laguna, Tenerife, Canary Islands, 38320 La Laguna, Spain

**Keywords:** alpha-Klotho, cirrhosis, prognosis, inflammation, lipid peroxidation

## Abstract

α-Klotho (Klotho) is an antiaging hormone with anti-inflammatory and antioxidative properties. Some studies suggest that Klotho increases in response to enhanced oxidative damage and inflammation. Alcoholism is a proinflammatory condition. The aim of this study was to analyze the relationship between Klotho and the serum levels of the inflammatory markers in alcoholic liver disease and to assess its prognostic value. We included 184 alcoholics and 35 age- and sex-matched controls. We determined the serum levels of Klotho, the tumor necrosis factor (TNF)-α, interleukin (IL)-6, IL-8, and malondialdehyde (MDA), and routine laboratory variables. Patients were followed-up with during 16 ± 18 months; 67 patients died. Klotho levels were higher among cirrhotics (with KW = 37.00 and *p* < 0.001) and were related to the Child–Pugh score (with KW = 15.96 and *p* < 0.001) and to the TNF-α (ρ = 0.28; *p* < 0.001) and MDA (ρ = 0.21; *p* = 0.006). The child’s groups were associated with mortality, both in the univariate (with the log-rank = 13.56, *p* = 0.001, Breslow = 12.33, and *p* = 0.002) and multivariate (with β = 0.43, *p* = 0.02, and OR = 1.53 (1.07–2.15)) analyses, also introducing Klotho and the TNF-α as dichotomic variables. However, the independent prognostic value of the Child’s groups was displaced by Klotho when only cirrhotics were considered; Klotho, over the median (574.4 pg/mL), was associated with higher mortality (with *p* = 0.04 and OR = 2.68 (1.06–6.84)). We conclude that Klotho is increased in liver cirrhosis. It is directly related to TNF-α, MDA, and to mortality in cirrhotics.

## 1. Introduction

Alcoholism may be viewed as an inflammatory condition [1]. Smoldering inflammation in patients with alcohol addiction mainly depends on increased oxidative damage generated by ethanol metabolism [2], pro-inflammatory and pro-oxidant effects of acetaldehyde and its adducts [3], and cytokine secretion by macrophages and Kupffer cells (among other liver cells) triggered by the increased translocation of both Gram+ and, especially, Gram- bacteria from the intestine to the portal blood [4]. The early cytokine response includes interleukin (IL)-1, IL-6, and the tumor necrosis factor (TNF)-α secretion, among other mediators [5]. These cytokines are able to increase the generation of reactive oxygen species (ROS) and induce the secretion of other inflammatory interleukins upon the activation of T-helper (Th) lymphocytes (Th-1 and Th-2 [6]). The ROS also activate the nuclear transcription factor, κ B (NF-κB), inducing the synthesis and secretion of more cytokines and closing a proinflammatory positive feed-back loop [7]. Intra- and extrahepatic porto-systemic shunts in liver cirrhosis increase the amount of intestinal bacteria that reach the specific immune system, leading to a more intense inflammatory response and oxidative lesion than those observed in non-cirrhotic alcoholics [8]. As described, oxidative damage is a key mediator of many harmful effects exerted by ethanol [3].

Klotho is a membrane-bound protein that may act as a receptor for the fibroblast growth factor 23 (FGF23). The cleavage of this membrane-bound Klotho by proteases, such as a disintegrin and metalloproteinase (ADAM) 10 and ADAM 17, generates the soluble Klotho protein, a multifunctional hormone with powerful anti-inflammatory and antioxidant effects [9,10], related to longevity in experimental models [11,12]. Indeed, Klotho blunts the activation of the transcription factor NF-κB and down-regulates the expression of the intercellular adhesion molecule and vascular cell adhesion molecule [13]; it decreases the TNF-α-induced secretion of IL-6 by endothelial cells [14] and inhibits the expression of the monocyte chemoattractant protein [15]. In addition to these anti-inflammatory actions, Klotho also up-regulates the expression of the catalase [16] and superoxide dismutase [17] and exerts other antioxidant effects acting on the NADPH- oxidase and angiotensin II [18]. Therefore, it is not surprising that low Klotho levels were related to mortality [19,20]. However, other authors have failed to find a relationship between Klotho and survival [21], and, even more, Abdelmalik et al. report that increased Klotho levels were independently related to mortality in patients with septic shock [22].

In previous reports, we have found that, despite its “protective” effect, Klotho levels were higher in cirrhotics and were related to liver function impairment [23]. We interpret that these results are perhaps in relation to a reactive increase of Klotho in response to inflammation, in accordance with the observations of Abdelmalik et al., who report that increased Klotho levels were related to mortality in patients with septic shock [22]. Alvarez-Cienfuegos et al. report higher Klotho levels in patients with rheumatoid arthritis, which were related to higher levels of the rheumatoid factor, anticitrullinated protein antibodies, and disease activity [24]. Also, in a very recent multicentric study on patients with systemic lupus erythematosus, Klotho levels were higher in patients with musculo-skeletal affectation and, globally, did not differ among the patients and controls [25]. Therefore, inflammation seems to play a role in raised Klotho levels in some instances. Based on this reasoning, the aim of the present study is to analyze the relationship of Klotho with proinflammatory cytokines (TNF-α, IL-6, and IL-8) and the lipid peroxidation products (Malondialdhyde = MDA) among alcoholics with or without liver cirrhosis, and its relationship with survival.

## 2. Materials and Methods

### 2.1. Patients and Controls

This study included a total of 184 patients (12 women), aged 58.94 ± 11.27 years, drinkers of 189 ± 128 g of alcohol per day for 32 ± 13 years, who were consecutively admitted to the internal medicine service of our hospital. Klotho was also determined to 35 controls of a similar age (56.40 ± 6.84 years; t = 1.79; *p* = 0.08) and sex (6 women, χ2 = 2.45; *p* = 0.12) χ2 = 3.10; *p* = 0.08).

All these patients were subjected to an ultrasound examination of the liver and spleen. A diagnosis of cirrhosis was established, combining ultrasound-derived data (liver with a heterogeneous texture with nodules, dilated portal vein, and splenomegaly) and clinical (ascites and encephalopathy) and laboratory data (prothrombin activity, albumin, and bilirubin). The study was reviewed and approved by the Institutional Review Board of our hospital (No. 2019-83).

Patients (besides 1) were followed-up during 16 ± 18 months (median = 7; interquartile range = 1–30 months). During this period, 67 patients died. The causes of death included liver failure in 14 cases; sepsis in 20 cases; neoplasia in 10 cases; vascular causes (including sudden death) in 4 cases; unknown in 19 cases.

### 2.2. Laboratory Testing

All the patients underwent a routine laboratory evaluation. The serum albumin, prothrombin activity, and serum bilirubin were recorded to assess liver function and to calculate the Child–Pugh’s score (Child’s score) [26]. This score includes the presence or not of ascitis and encephalopathy and the prothrombin activity, serum bilirubin, and serum albumin, assigning 1, 2, or 3 points to each of these items, according to severity, so that the score values ranged from 5 (fully compensated) to 15 (fully decompensated). Following well-established criteria, patients were further classified as Child A (5 or 6 points), B (7–9 points), and C (10–15 points). Other data, such as the mean corpuscular volume (MCV) and gamma-glutamyl transferase (GGT), were used in this study as “markers” of ethanol consumption and the C-reactive protein as a non-specific marker of inflammation.

After blood extraction, samples were placed in vacutainer tubes without an anticoagulant and allowed to clot (a maximum of 10 min at room temperature); then, they were centrifuged immediately at 3000 rpm at 4 °C for 5 min. After that, 200 µL of the serum was separated, aliquoted in an Eppendorf tube (only for MDA analysis), and stored, frozen, at −87 °C until analysis. All serum freezing samples at −87 °C were stored at a time not exceeding 1 month for analysis and were later analyzed in batches.

Klotho levels were determined by ELISA (Immuno-Biological Laboratories, Fujioka, Japan). The interassay and intrassay variation coefficients ranged from 2.9–11.4% and 2.7–3.5%, respectively. The percentage of recovery ranged from 84.7 to 97.5%. The sensitivity was 6.15 pg/mL, and for the cross-reactivity with other molecules, such as osteopontin, the platelet-derived growth factor, or the vascular endothelial growth factor, it was <0.1%. The TNF-α, IL-6, and IL-8 were determined by a Luminex^®^ Performance Assay (R&D Systems, Minneapolis, MN, USA), with a sensitivity of 0.29 pg/mL for the TNF-α, a sensitivity of 0.36 pg/mL for IL-6, and a sensitivity of 1.97 pg/mL for IL-8.

Serum MDA levels, referred to as the thiobarbituric acid-reactive substance (TBARS), were measured according to the method described by Kikugawa et al. [27], with some modifications. A volume sample of 0.2 mL was added to 0.2 mL of H3PO4 (0.2 M), and the colour reaction was initiated by the addition of 25 µL of a 0.11 M thiobarbituric acid (TBA) solution. Samples were placed in a 90 °C heating block for 50 min. After the samples were cooled, the TBARS (pink complex colour) were extracted with 0.4 mL of n-butanol. The Butanolic phase was separated by centrifugation at 6000× *g* for 10 min. Each one was placed in a 96 well plate and read at 535 nm in a microplate spectrophotometer reader (Spectra MAX-190, Molecular Devices, Sunnyvale, CA, USA). The calibration curve was prepared with authentic MDA standards. The detection limit of this assay was established in 0.079 µmol/L; the intra- and inter-assay of the coefficient of variations were 1.82% and 4.01%, respectively. The serum concentration of MDA was expressed in micromoles per liter. To avoid possible interferences of compounds that react or absorb at 532 nm, each sample was provided by our blank tube (sample without the TBA reagent), and the absorbance was subtracted from each sample tube [28]. Furthermore, in this assay, the use of butanol as the extracting agent of the complex of TBARS prevented many of these interferences [29].

### 2.3. Statistics

The Kolmogorov–Smirnov test was used to explore if the variables showed a normal distribution or not. A Student’s *t*-test was used with the variables with a normal distribution, whereas non-parametric tests, such as the Mann–Whitney’s U (Z) test, Kruskal–Wallis (KW), and Spearman’s (ρ) correlation tests, were used when the variables included in any of these analyses showed a non-parametric distribution. Qualitative variables were compared by means of a chi-square test. Survival was analyzed by means of Kaplan–Meier curves, comparing patients who died during the observation period with those who survived using a log-rank (LR) and Breslow tests. We also performed a stepwise Cox regression analysis, introducing variables that showed significant differences between survivors and non-survivors in the univariate analysis. Non-parametric variables, such as cytokines or MDA, were dichotomized according to the median values or, in the case of the TNF-α, according to detection level. All these analyses were performed with SPSS software (15.0) (Chicago, IL, USA).

## 3. Results

### 3.1. Liver Function

**Figure 1:**
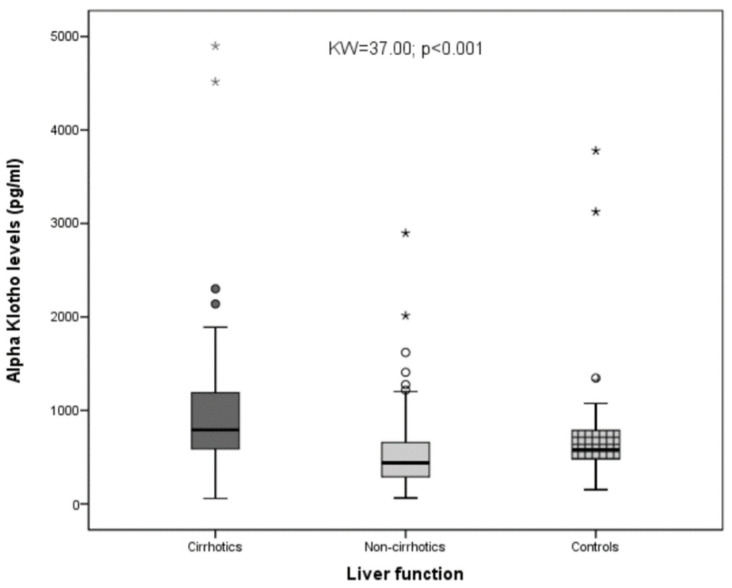
Differences in the serum Klotho values among the cirrhotics, non-cirrhotics, and healthy controls. As shown, statistically significant differences were observed when the three groups were compared (KW = 37.00; *p* < 0.001), * and ° = represent extreme values.

Among the whole sample, we observed that cirrhotics showed significantly higher Klotho values than non-cirrhotics. Non-cirrhotics showed significantly lower Klotho values when compared with the controls (with *p* < 0.05 with the SNK test). Globally, the differences were highly significant (with KW = 37.00 and *p* < 0.001; Figure 1).

**Figure 2:**
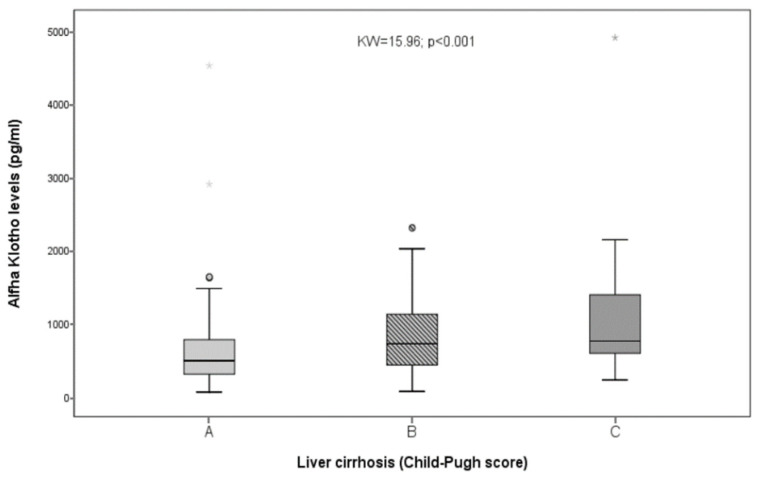
Differences in Klotho values among Child A, B, and C patients. As shown, statistically significant differences were observed when the three groups were compared (KW = 15.96; *p* < 0.001), ° = represent extreme values.

Klotho was significantly related to prothrombin activity (with ρ = −0.39 and *p* < 0.001), albumin (with ρ = −0.15 and *p* = 0.047), and bilirubin (with ρ = 0.26 and *p* < 0.001) but not to serum creatinine (with ρ = −0.03 and *p* = 0.72; NS). Marked differences were observed when Klotho was compared among the three Child’s groups (with KW = 15.96 and *p* < 0.001; Figure 2), and the patients belonging to the Child’s B or C groups showed significantly higher Klotho values (Z = 3.11; *p* = 0.002) than patients belonging to the Child A group. Klotho was also related to platelet count (ρ = −0.39; *p* < 0.001). Therefore, the Klotho values were higher among cirrhotics and were related to liver function impairment. 

### 3.2. Klotho and Inflammation

Significant relationships were observed between Klotho and the TNF-α (with ρ = 0.28 and *p* < 0.001) and between Klotho and MDA (with ρ = 0.21 and *p* = 0.006) but not between Klotho and IL-6 or IL-8. The TNF-α values were significantly lower among patients with Klotho values below the median than among patients with Klotho values over the median (with Z = 2.85 and *p* = 0.004). Also, the MDA values were significantly higher among patients with α-Klotho values over the median (with Z = 3.012 and *p* = 0.003). Therefore, Klotho levels were associated with increased lipid peroxidation and increased serum TNF-α values.

As shown in Table 1, cirrhotics showed higher IL-8 and higher MDA levels than non-cirrhotics. However, no differences were observed regarding the TNF-α and IL-6 levels when comparing cirrhotics and non-cirrhotics, and, also, we failed to find any association among detectable TNF-α levels and liver cirrhosis (with χ2 = 1.18 and *p* = 0.28 (NS). Moreover, the TNF-α levels were not related to albumin, prothrombin, bilirubin, or the Child–Pugh score. This was not the case for IL-8, which was significantly higher in cirrhotics (with Z = 4.06 and *p* < 0.001), especially in the more decompensated ones (with KW = 11.9 and *p* = 0.003 when IL-8 was compared among the three Child’s groups) and closely related to the prothrombin activity (with ρ = −0.21 and *p* = 0.006) or the platelet count (with ρ = −0.30 and *p* < 0.001). A somewhat weaker association with the liver function was observed with IL-6. When classified as a dichotomic variable, according to the median values, the IL-6 levels over the median were significantly associated with liver cirrhosis (with χ2 = 9.77 and *p* = 0.002), and the IL-6 values were inversely related to the prothrombin activity (with ρ = −0.30 and *p* < 0.001). In addition, IL-8 (with Z = 3.87 and *p* < 0.001) and MDA (with Z = 3.98 and *p* < 0.001) were significantly higher among patients with IL-6 values over the median. The TNF-α values were also higher in this group but did not reach a statistical significance (with Z = 1.60 and *p* = 0.11).

Relationships between MDA and liver function were stronger than those observed between cytokine levels and liver function. The MDA values over the median were associated with liver cirrhosis (χ2 = 8.36 and *p* = 0.004), and the MDA values were strongly associated with liver function impairment (ρ = −0.54; *p* < 0.001 with prothrombin; ρ = −0.16; *p* = 0.042 with albumin; KW = 14.95; and *p* < 0.001 when compared among the three Child’s groups, where higher values were observed among Child’s C patients) and with the platelet count (where ρ = −0.31 and *p* < 0.001). Also, significant relationships were observed between the MDA levels and IL-6 (with ρ = 0.31 and *p* < 0.001) and IL-8 (with ρ = 0.49 and *p* < 0.001) but not with the TNF-α (with ρ = 0.06 and *p* = 0.46). Therefore, lipid peroxidation is increased in liver cirrhosis and is related to liver function impairment. MDA is also related to the proinflammatory cytokines IL-6 and IL-8. Liver cirrhosis was significantly associated with IL-6 and IL-8 over the median but not with the TNF-α values.

### 3.3. Survival

**Figure 3:**
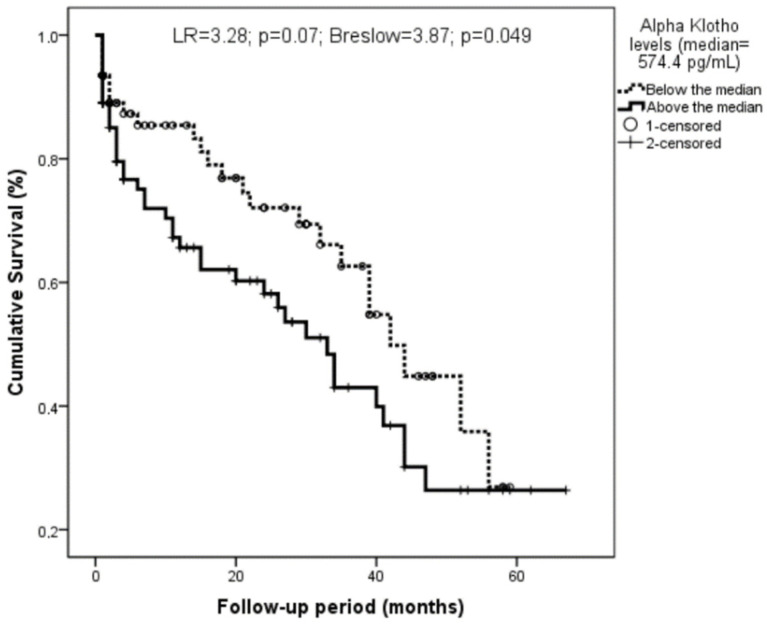
Relationship of Klotho with survival among the whole sample. Klotho levels were classified according to the median (574.4 pg/mL).

Sixty-seven patients died during the follow-up period. Both age (with Z = 2.52 and *p* = 0.012) and Klotho (with Z = 2.46 and *p* = 0.014) were higher among those who died. Those patients with Klotho levels over the median showed a marginally significant trend to a higher mortality (with LR = 3.28, *p* = 0.07, Breslow = 3.87, and *p* = 0.049; Figure 3).

**Figure 4:**
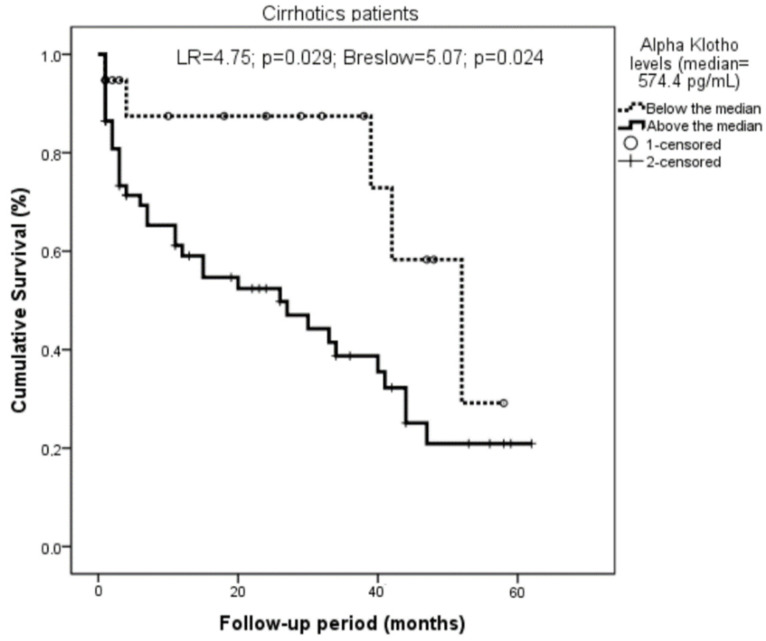
Relationship of Klotho with survival among the cirrhotics. Klotho levels were classified according to the median (574.4 pg/mL). Cirrhotic patients with Klotho values above the median showed higher mortality (LR = 4.75; *p* = 0.029; Breslow = 5.07; *p* = 0.024).

This relationship was highly significant when only the cirrhotics were considered (with LR = 4.75, *p* = 0.029, Breslow = 5.07, and *p* = 0.024; Figure 4).

**Figure 5:**
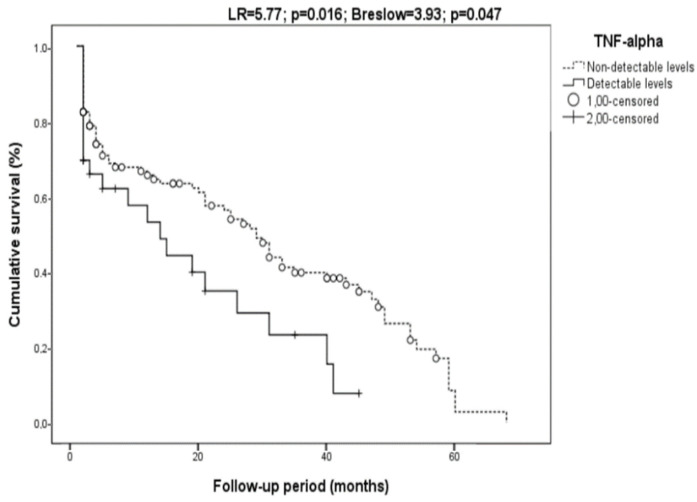
Relationship of the TNF-α with survival. The TNF-α levels were classified according to detectable or non-detectable levels. Patients with detectable TNF-α values showed higher mortality (LR = 5.77; *p* = 0.016; Breslow = 3.93; *p* = 0.047).

Survival was also marginally related to MDA (with LR = 2.87, *p* = 0.09, Breslow = 3.75, and *p* = 0.05) but not in cirrhotics only. The TNF-α was also related to mortality (with LR = 5.77, *p* = 0.016, Breslow = 3.93, and *p* = 0.047 (Figure 5)) but not in cirrhotics (with LR = 1.00, *p* = 0.32, Breslow = 2.13, and *p* = 0.15).

**Figure 6:**
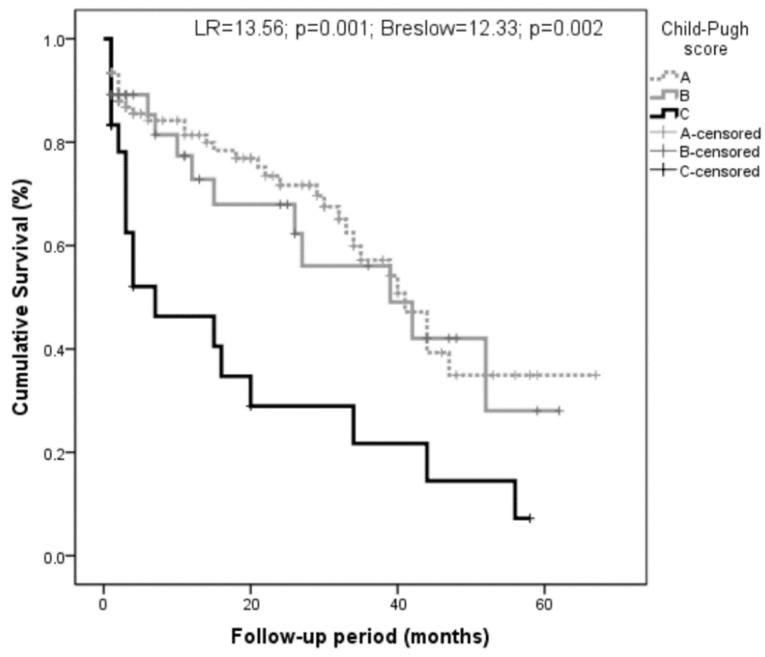
Relationship of the Child’s groups with survival. Patients with worse liver function (Child C) had higher mortality (LR = 13.56; *p* = 0.001; Breslow = 12.33; *p* = 0.002).

The Child groups were strongly associated with mortality (with LR = 13.56, *p* = 0.001, Breslow = 12.33, and *p* = 0.002; Figure 6). Age was not associated with mortality (with LR = 0.18, *p* = 0.67, Breslow = 0.16, and *p* = 0.69).

The Child–Pugh groups had the only variable that showed an independent relationship with mortality (with beta = 0.43, *p* = 0.02, and RR = 1.53 (1.07–2.15) when assessed by the Cox regression model, also introducing Klotho and the TNF-α as dichotomic variables. Performing the same analysis only in cirrhotics, we observed that Klotho classified in the medians displaced the Child’s score, where α-Klotho, over the median (574.4 pg/mL), was associated with higher mortality (with *p* = 0.04 and RR = 2.68 (1.06–6.84)).

## 4. Discussion

We have shown that Klotho levels are higher among cirrhotics [23]. This has been confirmed in this work, which includes new patients that behave in a similar fashion to those previously reported. Klotho raises among cirrhotics and is related to variables associated with disease progression. This is in agreement with the observations of Prystupa et al., who report a trend with higher Klotho values among Child’s B or C patients [30], although data derived from other studies suggest an opposite effect. Rao et al. reported a lower lipid accumulation in the liver of Klotho-treated obese mice compared to the controls [31]. Also, in a recent study, the Klotho expression in hepatocellular carcinoma was a marker of a good prognosis [32]. In addition, some polymorphisms of the *Klotho* gene have been associated with a protective effect against hepatic steatosis in patients with non-alcoholic fatty liver disease [33].

In this study, Klotho levels were associated with liver function impairment and higher mortality and also with MDA and TNF-α levels. All these data suggest that Klotho levels increase in patients with alcoholic liver disease, keeping a relationship with liver function impairment. On the other hand, liver function impairment is related to increased cytokine (with the exception of the TNF-α) and MDA levels, a finding shared by the vast majority of authors involved in research related to the alcoholism-associated inflammatory response. As commented, alcoholism is associated with increased intestinal permeability that leads to increased amounts of Gram- (and Gram+) bacteria in the portal blood. These bacteria activate liver macrophages and Kupffer cells, leading to increased cytokine secretion, especially the TNF-α and IL-6, and the production of ROS. This pathway importantly contributes to the proinflammatory status that can be observed in alcoholics. In liver cirrhosis, the distortion of the vascular architecture facilitates the escape of intestinal bacteria to the systemic circulation, stimulating many cells of the innate immune system that can secrete proinflammatory cytokines, such as the TNF-α. Therefore, theoretically, cytokine levels are higher, and the ROS production is increased among cirrhotics. In this study, we observed increased MDA in cirrhotics and also increased IL-6 and Il-8, but not increased TNF-α values. The lack of differences between cirrhotics and non-cirrhotics in the TNF-α levels is difficult to explain. An unexpected large proportion of patients (78%) showed a TNF-α below the detection levels, but all the TNF-α, IL-6 (20% of the cases below the detection level), IL-8 (in only one case below the detection levels), Klotho (in none of the cases below the detection level), and MDA (in none of the cases below the detection level) were determined in the same blood sample. In summary, Klotho, MDA, IL-8, and IL-6 behave similarly in patients with alcoholic liver disease, being more or less intensely related to disease progression and liver function impairment. This was not observed with the TNF-α, in contrast with the results of most studies [34].

However, despite the lack of differences between cirrhotics and non-cirrhotics regarding TNF-α values, Klotho levels were directly related to the TNF-α values. There may be a theoretical basis to explain this relationship. The TNF-α may repress Klotho mRNA transcription [35]. The Klotho gene codifies for a transmembrane protein that may be attacked by membrane-bound α secretases, including ADAM 10, ADAM 17, and the β amyloid precursor protein cleavage enzyme-1 (BACE 1). These secretases promote the shedding of the soluble protein Klotho, a process that is activated by insulin (acting on ADAM 10 and ADAM 17 [36]) but also, at least theoretically, by the TNF-α, that induces the ADAM 10 expression [37]. Therefore, the TNF-α may exert a dual effect on soluble Klotho, reducing the transcription of the transmembrane molecule but increasing the shedding of soluble Klotho from this molecule by increasing the ADAM 10 expression. This complicated pathway might explain our results regarding the significant association between high TNF-α values and high Klotho levels and the association between Klotho and other variables related to inflammation, such as MDA levels (given the close relationship between inflammation and ROS production). However, according to the present study, this interpretation is merely speculative because the finding of a significant correlation among two variables does not imply that there exists a causal relationship among them, but the results of this observational research are in accordance with the results of Abdelmalik et al. in septic patients [22] and with those observed by Álvarez-Cienfuegos et al. in patients affected with rheumatoid arthritis [24].

Klotho has been considered a protective hormone, exerting antioxidant effects [18] and depressing cytokine expression [38]. These actions may explain the anti-senescence effects of Klotho [39], providing theoretical support to the findings of the inverse relationship between Klotho and brain atrophy. The antioxidant effect explains the vasculo-protective effect exhibited by Klotho [40], lending support to the finding of lower Klotho values observed among alcoholics with left ventricular hypertrophy or atrial fibrillation and findings reported by several authors and also by ourselves in a previous report [23]. However, in the present study, increased Klotho keeps a relationship with increased MDA levels. As with the relationship of Klotho with the TNF-α, this result may be interpreted as derived from a compensatory increase of Klotho levels in a context of an increased ROS production (as is the case of alcoholism and, especially, alcoholic cirrhosis). This hypothetical compensatory increase of Klotho levels in response to inflammation has also been argued by Álvarez-Cienfuegos et al. to explain the increased levels in patients with rheumatoid arthritis, a major inflammatory disease [24]. The hypothetical reactive increase of Klotho in liver cirrhosis may explain the counterintuitive relationship of Klotho with survival that is especially marked in cirrhotics. This result is fully in accordance with the results of Abdelmalik et al. in septic patients and probably obeys a similar mechanism. Abdelmalik et al. explain their findings on the basis of a compensatory increase of Klotho in response to inflammation [22], but, as commented, in this observational study, we are unable to discern the mechanisms involved in the reported increase in Klotho in patients with alcoholic liver disease and why increased Klotho is related to mortality.

In addition to the shortage imposed by the lack of differences in the TNFα values among cirrhotics and non-cirrhotics, there are other limitations of this study. The diagnosis of cirrhosis was made on clinical/ultrasonographic features. This may constitute a shortage of our study. Moreover, strictly speaking, the Child’s score should only be applied to cirrhotics, but for the sake of uniformity, we also used it for non-cirrhotic patients since it is an excellent tool to globally assess the derangement of the liver function and progression of liver disease. Following Prystupa et al. [30], we further grouped the patients into those with deranged liver function (Child B and C) and those with preserved liver function (Child A). The finding of the marked differences between both groups and the significant correlations of Klotho with several variables, such as prothrombin activity, bilirubin, and platelets, is strong enough to conclude that Klotho is related to liver disease progression among patients with alcohol use disorder.

## 5. Conclusions

We conclude that Klotho is increased in liver cirrhosis, being related to the TNF-α, lipid peroxidation, and especially to liver function impairment. Possibly, this association may explain the counterintuitive results regarding the relationship of Klotho with mortality, something observed not only in the whole group of alcoholics but also, especially, among cirrhotics. Further studies are needed to assess the behavior of Klotho in other instances characterized by an intense inflammatory response and to discern the mechanisms explaining the (possible) increase of Klotho levels in these conditions.

## Figures and Tables

**Table 1:** Comparison of the variables used in this study among cirrhotics and non-cirrhotics.

	Cirrhotics (79)	Non-Cirrhotics (105)	T (Z); χ2
Sex (men/women)	73/6	99/6	χ2 = 0.04; *p* = 0.83 (NS)
Age (years)	60.05 ± 9.87	58.10 ± 12.20	T = 1.16; *p* = 0.25 (NS)
Daily ethanol (g)	194 ± 101 200 (120–243)	183 ± 146 147 (96–228)	Z = 1.67; *p* = 0.10 (NS)
Years of addiction	32 ± 13	33 ± 14	T = 0.37; *p* = 0.71 (NS)
MCV (fL)	101.14 ± 10.22 100.60 (96.45–104.00)	99.82 ± 6.17 100.60 (95.30–107.70)	Z = 0.76; *p* = 0.45 (NS)
GGT (U/L)	348.53 ± 564.90 197.00 (71.00–390.00)	209.29 ± 264.31 101.00 (54.00–243.50)	Z = 2.45; *p* = 0.014
Klotho (pg/mL)	967.98 ± 769.34 792.00 (580.60–1192.80)	533.05 ± 410.11 439.40(289.35–660.64)	Z = 5.76; *p* < 0.001
IL-6 (pg/mL) (*)	14.68 ± 16.02 10.18 (4.08–18.23)	15.21 ± 23.96 4.85 (0.92–19.64)	Z = 1.73; *p* = 0.08 (NS)
IL-8 (pg/mL) (**)	99.30 ± 170.42 40.67 (22.65–100.00)	49.10 ± 181.98 18.41 (11.36–30.97)	Z = 4.06; *p* < 0.001
TNF-α (pg/mL) (***)	5.27 ± 5.85 5.00 (1.59–5.00)	7.72 ± 11.97 5.00 (2.75–5.00)	Z = 1.48; *p* = 0.14 (NS)
CRP (mg/L) (****)	26.81 ± 38.12 14.40 (4.30–33.00)	31.04 ± 56.24 11.90 (4.20–33.13)	Z = 0.22; *p* = 0.82 (NS)
MDA (µmol/L) (*****)	4.32 ± 4.10 2.92 (1.93–5.30)	2.75 ± 2.96 1.88 (1.26–2.84)	Z = 3.93; *p* < 0.001
Prothrombin activity (%)	66.37 ± 20.65 66.00 (53.00–83.00)	87.30 ± 14.08 89.00 (79.00–100.00)	Z = 6.87; *p* < 0.001
Albumin (g/dL)	3.50 ± 0.80 3.50 (3.00–4.00)	3.76 ± 0.60 3.80 (3.40–4.20)	T = 2.43; *p* = 0.016
Bilirubin (mg/dL)	3.41 ± 4.06 2.00 (1.00–4.20)	1.33 ± 1.38 1.00 (1.00–2.00)	Z = 5.88; *p* < 0.001
Platelet count (/µL)	136,784 ± 91,302 118,000 (68,000–167,000)	234,298 ± 116,588 213,000 (149,500–292,500)	Z = 6.39; *p* < 0.001
Serum creatinine (mg/dL)	1.05 ± 0.78 0.80 (0.63–1.10)	0.84 ± 0.40 0.76 (0.61–0.91)	Z = 1.32; *p* = 0.18 (NS)

Data are given as mean ± standard deviation and as median and (interquartile range) when the variables showed a non-parametric distribution. * *n* = 150 (66 cirrhotics); ** *n* = 101 (42 cirrhotics); *** *n* = 152 (66 cirrhotics); **** *n* = 179 (77 cirrhotics); ***** *n* = 171 (70 cirrhotics). Cytokine, MDA, Klotho, cirrhosis, and survival data are shown in the Supplementary Materials.

## Data Availability

The data presented in this study are available on request from the corresponding author.

## Supplementary Materials

The following supporting information can be downloaded at: https://www.mdpi.com/article/10.3390/biom12081151/s1. Table S1: Cytokine, MDA, Klotho, cirrhosis and survival data.
