# Supplementary material for: Klotho Levels and Their Relationship with Inflammation and Survival among Alcoholic Patients
Source: Biomolecules. 2022 Aug 20;12(8):1151. doi: 10.3390/biom12081151 (PMC9405938; doi:10.3390/biom12081151)
Supplement: Supplementary file 1 [file biomolecules-12-01151-s001.zip › biomolecules-1827526-SI.pdf]

Supplementary Materials Table S1: Cytokine, MDA, Klotho, cirrhosis and survival data.

	Klotho (pg/mL)	MDA* (µmol/L)	TNF-α** (pg/mL)	IL-6*** (pg/mL)	Il-8**** (pg/mL)	Cirrhosis (n=79)	Survival
1	708.60	1.29	BD	2.96		NO	A
2	777.10	2.15	BD	10.30		YES	A
3	355.80	1.09	BD	BD		NO	A
4	523.80	2.38				NO	D
5	624.60	1.55	BD	6.28		YES	D
6	128.50	1.49	BD	4.13	19.86	NO	D
7	57.60	3.47	BD	57.79	114.49	YES	A
8	342.00	5.08				NO	A
9	521.10	.38	BD	3.76		NO	A
10	454.50	1.14	BD	BD		NO	A
11	1735.90	6.15	BD	9.38	138.91	YES	A
12	568.20	.88	BD	1.82		NO	A
13	164.10	2.77				NO	A
14	528.50	1.82	BD	22.80	44.33	YES	D
15	133.00	1.26	3.86	BD	19.86	NO	A
16	427.50	4.02	BD	16.28	56.45	YES	D
17	406.50	.20	BD	BD		NO	D
18	273.10	2.93	BD	BD		YES	A
19	1354.80	6.44				YES	D
20	772.20	.98	BD	BD		NO	A
21	690.30	3.09	BD	8.63	74.15	YES	D
22	319.00	1.23	BD	1.58		NO	A
23	236.70	2.56	9.89	29.50	63.20	NO	D
24	289.59	2.93	3.06	19.73	23.89	NO	A
25	736.50	.74	BD	BD		NO	D
26	606.00	1.47				NO	A
27	773.40	2.62	BD	9.84		YES	D
28	351.20	1.28	1.82	BD	27.63	NO	A
29	521.10	1.87	BD	BD		NO	D
30	360.50	3.51				NO	D
31	486.20	2.53	BD	12.49	78.84	YES	A
32	643.50	2.45	BD	9.48		YES	D
33	792.00	7.02	7.94	2.15	37.00	YES	D
34	636.60	5.23	23.70	16.60	293.00	YES	D
35	309.80	.92	BD	32.81	24.41	NO	A
36	335.40	8.09				NO	A
37	609.10	2.16				NO	A
38	190.65	4.91	BD	5.58	21.84	NO	A
39	1165.50	.50	BD	37.34		NO	D
40	2301.00	3.36				YES	D
41	210.30	1.30	BD	1.45	14.30	YES	A
42	300.60	1.76	BD	7.55	17.00	NO	A
43	439.40	1.46	6.18	18.88	48.92	NO	A
44	523.80	5.59	28.20	43.12	29.28	NO	D
45	291.40	2.71	20.43	87.97	68.06	NO	D
46	368.40	3.63	3.46	11.53	187.39	YES	A
47	486.20	1.88	BD	21.98	10.17	NO	A
48	355.20	3.65	BD			NO	A
49	302.70	5.35	BD			NO	A
50	342.00	2.26	BD	17.53	22.86	YES	A
51	1047.90	2.90	BD	9.84		YES	A
52	144.30	3.75	BD	28.34	19.86	NO	A
53	1185.84					YES	A
54	291.40	1.14	BD	BD	12.59	YES	D

55	687.00	3.32				NO	A
56	245.80	2.02	65.72	100.84	46.93	NO	A
57	596.40	2.61	BD	16.40	22.00	YES	D
58	510.00	1.28				NO	A
59	552.10	2.36	BD	40.53	13.87	YES	A
60	690.30	1.96	BD	87.66	29.84	YES	A
61	709.50	3.55	4.65	16.91	1008.49	YES	D
62	483.00	1.24	BD	BD		NO	A
63	845.43	2.33				YES	D
64	918.66	1.09				YES	D
65	623.40	4.00	6.56	7.87	11.36	NO	A
66	460.20	1.54	BD	BD	18.41	NO	A
67	603.90	4.31	BD	31.52	175.56	YES	D
68	273.10	2.79	BD	71.72	32.69	NO	A
69	223.50	17.34	1.82	BD	223.48	YES	D
70	464.10		BD	43.80		NO	A
71	1471.20		BD	39.92		YES	D
72	407.70	3.67	BD	10.40	59.99	YES	A
73	186.60	1.57	5.76	5.12		NO	A
74	66.00	2.48	BD	15.90	60.71	NO	A
75	492.60	.82	BD	.99		NO	D
76	825.90	5.55	12.77	60.82	280.89	YES	D
77	580.60	5.51	3.06	27.23	445.83	YES	A
78	198.90		BD	3.98		NO	A
79	381.60	3.41	16.98	1.53	17.47	NO	D
80	828.60	1.71				YES	D
81	656.80	2.06	BD	15.39	62.88	NO	A
82	279.30	2.98	BD	.99		YES	A
83	547.40	1.16	BD	BD	14.74	NO	A
84	200.40	3.45	BD	5.86	15.63	NO	D
85	342.00	5.93	BD	BD	47.59	YES	A
86	492.60	2.94	BD	3.18		YES	A
87	741.00	1.61				YES	A
88	273.10	2.89	BD	8.18	28.17	YES	A
89	111.00	2.11	9.89	24.29	21.34	NO	A
90	204.90	2.42	BD	81.28	23.37	NO	A
91	289.20	1.79	BD	46.28		NO	A
92	315.90	4.00				NO	A
93	365.10	1.08	BD	BD	12.18	NO	D
94	911.10		BD	27.18		YES	D
95	1138.50	.80	BD	20.32		YES	D
96	714.90	5.59	10.26	41.67	93.53	YES	D
97	652.00	2.61	BD	BD	50.93	NO	D
98	374.70	1.88				NO	A
99	688.80	1.82				YES	A
100	243.60	1.26				YES	A
101	358.02	1.12	BD	2.26		NO	A
102	397.50	3.31	8.32	.99		NO	A
103	346.60	1.72	BD	8.33	14.30	NO	A
104	232.10	1.06	4.25	11.67	14.74	NO	A
105	1318.80	5.14	BD	11.20		YES	D
106	938.73	3.95	BD	10.06		YES	A
107	459.00	2.58				NO	A
108	773.40	.84	BD	20.56		YES	D
109	1216.80	7.74				NO	D
110	407.70	6.79	15.94	27.79	1411.65	NO	A
111	514.40	1.90	BD	4.32	17.94	NO	D
112	623.61	1.05	BD	15.90	44.97	YES	D

113	63.12	1.62	BD	19.37	30.97	NO	A
114	1309.10	4.13	BD	27.12	24.94	YES	A
115	84.10	.94	BD	36.12	8.65	NO	D
116	444.90	.78	BD	BD	29.10	YES	A
117	223.50	1.20				NO	A
118	717.90	.90	BD	BD		NO	A
119	2014.80	13.78	BD	24.86		NO	D
120	434.80	1.44	47.19	3.54	136.97	NO	A
121	430.10	1.26	BD		18.41	NO	D
122	289.50	2.27	9.89	18.76	22.35	NO	A
123	186.90	1.51	BD	2.28		NO	A
124	1202.50	5.37	30.86	20.33	39.90	NO	A
125	66.00	1.52	BD	11.81	19.37	YES	D
126	200.40	3.39				NO	D
127	806.10	9.80				YES	D
128	828.60		BD	5.58		YES	D
129	894.00	2.02	BD	26.67	25.47	YES	A
130	714.90	19.90				YES	D
131	4516.13	2.53	BD	10.58	7.99	YES	D
132	682.20		BD	14.92	100.00	YES	A
133	216.90	1.71	BD	BD	11.90	NO	A
134	203.70	1.50	BD	4.58	8.67	NO	D
135	1008.00	6.28	BD	10.96	21.30	NO	D
136	243.48	1.87	BD	BD	6.48	NO	D
137	1211.80	1.76	BD	8.04	16.50	YES	D
138	963.60	1.78	BD	BD	2.91	NO	A
139	433.15	1.59	BD	BD	8.08	NO	A
140	2896.45	1.97			3.16	NO	A
141	585.83	5.25			58.90	NO	A
142	730.10	6.30	BD	BD	73.40	YES	A
143	1407.66	2.65	BD	6.04	12.50	NO	A
144	815.58	2.20	BD	BD	75.90	YES	D
145	1165.68					YES	A
146	826.68	11.48	BD	BD	100.00	YES	A
147	1148.39	1.31	BD	4.02	9.59	YES	A
148	807.25	1.86	20.90	8.22	24.30	YES	A
149	267.24	18.05	BD	129.34	48.10	NO	A
150	708.21	2.36	74.58	6.22	80.60	NO	A
151	372.25	2.44	BD	28.04	13.60	NO	A
152	952.32	2.33	BD	2.36	8.28	NO	A
153	1322.41	2.14	BD	BD	BD	NO	A
154	832.24	2.29	BD	BD	15.00	YES	A
155	664.48	2.81	BD	BD	2.46	NO	A
156	963.60	1.69	25.26	5.32	5.84	NO	A
157	1068.31	1.16	BD	6.22	8.13	NO	A
158	955.14	1.18	BD	1.80	9.25	NO	A
159	377.53	2.53	BD	4.20	13.90	NO	A
160	1619.92	2.87	BD	5.86	27.40	NO	A
161	1051.28	2.33	BD	BD	6.92	NO	A
162	735.58	5.18	BD	6.22	4.21	YES	A
163	1797.04	19.10	BD	46.16	25.50	NO	A
164	719.16	16.74	BD	5.12	30.60	YES	D
165	1275.64	9.04	BD	5.46	33.20	YES	D
166	1425.44	1.46	BD	1.44	12.00	NO	A
167	1659.00	1.95	8.06	5.32	32.70	YES	A
168	736.50	2.78	BD	34.54		YES	D
169	819.60	.72	BD	3.54		NO	A
170	558.00	3.38	BD	9.02		1.00	A

171	671.40	1.18	6.60	2.28		NO	D
172	502.20	1.18	11.02	4.22		NO	A
173	2139.30	1.73	BD	6.16		NO	A
174	1120.50		40.84	14.10		YES	D
175	1192.80					YES	A
176	4899.90	2.03	BD	6.28		YES	A
177	1890.60	18.54	BD	10.86		NO	A
178	530.40	6.47	BD	21.50		YES	D
179	1336.80		BD	9.84		NO	A
180	549.60		BD	4.10		YES	D
181	1587.60		BD	13.52		NO	D
182	1630.20	1.17				YES	D
183	1437.50	4.58	BD	3.64		YES	A
184	164.40	8.85		10.64		YES	D

BD: below detection level. A: Alive. D: died. *n=171; **n=152; ***n=150; ****n=101.
